# Effect of Pre- and Postoperative Phenylbutazone and Morphine Administration on the Breathing Response to Skin Incision, Recovery Quality, Behavior, and Cardiorespiratory Variables in Horses Undergoing Fetlock Arthroscopy: A Pilot Study

**DOI:** 10.3389/fvets.2015.00058

**Published:** 2015-11-23

**Authors:** Clara Conde Ruiz, Inga-Catalina Cruz Benedetti, Isabelle Guillebert, Karine Genevieve Portier

**Affiliations:** ^1^Anaesthesiology, Pôle Equin, VetAgro Sup, Lyon University, Marcy L´Etoile, France; ^2^CarMeN Laboratory, INSERM UMR-1060, University of Lyon, Lyon, France

**Keywords:** horses, noxious stimulus, breathing response, analgesia, opioids

## Abstract

This prospective blinded randomized study aimed to determine whether the timing of morphine and phenylbutazone administration affects the breathing response to skin incision, recovery quality, behavior, and cardiorespiratory variables in horses undergoing fetlock arthroscopy. Ten Standardbred horses were premedicated with acepromazine (0.04 mg kg^−1^ IM) and romifidine (0.04 mg kg^−1^ IV). Anesthesia was induced with diazepam (0.05 mg kg^−1^) and ketamine (2.2 mg kg^−1^) IV at T0. Horses in group PRE (*n* = 5) received morphine (0.1 mg kg^−1^) and phenylbutazone (2.2 mg kg^−1^) IV after induction and an equivalent amount of saline after surgery. Horses in group POST (*n* = 5) received the inversed treatment. Anesthesia was maintained with isoflurane 2% in 100% oxygen. Hypotension (mean arterial pressure <60 mmHg) was treated with dobutamine. All horses breathed spontaneously. Dobutamine requirements, respiratory rate (*f*_R_), heart rate (HR), mean arterial blood pressure, end-tidal CO_2_, inspired (_i_) and expired (_e_) tidal and minute volume (*V*_T_ and ${\dot{V}}_{\text{E}}$), inspiratory time (IT), and the inspiratory gas flow (*V*_Ti_/IT) were measured every 5 min. Data were averaged during four 15 min periods before (P1 and P2) and after the incision (P3 and P4). Serial blood–gas analyses were also performed. Recoveries were unassisted, video recorded, and scored by three anesthetists blinded to the treatment. The postoperative behavior of the horses (25 demeanors), HR, and *f*_R_ were recorded at three time points before induction (T0–24 h, T0–12 h, and T0–2 h) and six time points after recovery (TR) (TR + 2, 4, 6, 12, 24, 48 h). Data were compared between groups using a Wilcoxon test and within groups using a Friedman test or a Kruskal–Wallis signed-rank test when applicable. Tidal volumes (*V*_Te_ and *V*_Ti_) were higher in PRE than in POST during all the considered periods but the difference between groups was only significant during P2 (*V*_Te_ in mL kg^−1^ in PRE: 13 [9, 15], in POST: 9 [8, 9], *p* = 0.01). None of the other variables were significantly different between and within groups. Under our experimental conditions, skin incision did not affect respiratory variables. Administration of pre- versus postoperative phenylbutazone and morphine did not influence recovery quality, HR, *f*_R_, or animal behavior.

## Introduction

Surgery performed in horses undergoing general anesthesia induces nociception which may affect the quality of recovery (1), the most critical phase of equine anesthesia (2). Early treatment of nociception with appropriate multimodal analgesia is therefore mandatory to improve the outcome of equine surgeries. Arthroscopy, in particular, has been used as a pain model in horses due to the fact that it can induce a moderate degree of postoperative pain requiring an optimal pain management (3, 4).

The identification of markers of nociception during the maintenance of anesthesia is important because it could lead to an early treatment of nociceptive stimulation.

In anesthetized human patients, some studies demonstrated that noxious stimuli affect respiratory variables (5–8). The ventilatory response to skin incision during anesthesia is an increase in mean inspiratory flow rate, defined as the amount of air the patient inspires (*V*_Ti_) by the time the inspiration lasts (IT): *V*_Ti_/IT. This is the result of an increase in tidal volume (*V*_T_) without changes in respiratory frequency. This was observed over the first breaths following this stimulus and it rapidly reverted to pre-incision values in patients anesthetized with halothane (5), enflurane (7), or with a propofol–alfentanil infusion (8). These studies suggest that *V*_Ti_/IT and *V*_T_ can be used as markers of nociception.

In veterinary medicine, we did not find similar studies which point to a possible relationship between nociception and respiratory drive under anesthesia. To the authors’ knowledge, the breathing response to skin incision has not been studied in anesthetized horses.

Non-steroidal anti-inflammatory drugs (phenylbutazone) and opioids (morphine) are commonly used as part of the analgesic protocol in equine patients undergoing arthroscopy. Opioids may have a variable influence on respiration (9). When associated to alpha-2 agonists, morphine produced respiratory depression (fall in PaO_2_ and rise in PaCO_2_) (10), whereas, when given as an intravenous (IV) bolus during general anesthesia, it did not affect *V*_T_, *f*_R_, or PaCO_2_, suggesting a lack of significant centrally induced respiratory depression (11). As opioids may influence the breathing response to noxious stimuli, their inclusion in the anesthetic protocol may question its efficiency as a marker of nociception.

In contrast, phenylbutazone does not seem to have an effect on respiration. This drug did not affect cardiorespiratory variables in healthy standing horses that received a combined administration of phenylbutazone and romifidine in comparison to romifidine alone (12).

The timing of analgesic administration may also affect the breathing response to skin incision. In addition, due to the subsequent surgery, a more severe postoperative pain may occur. The effects of preemptive analgesia on postoperative pain, behaviors, and particularly on recovery quality in horses, remain to be studied (13). Phenylbutazone, when given to horses undergoing arthroscopy preoperatively, seemed to have a postoperative analgesic benefit but altered the recovery score (including the number of attempts to stand, ataxia, and excitement as criteria) in comparison to the placebo (14). Although controversial, several studies demonstrated that morphine administered as preoperative treatment improved the quality of recovery (15, 16).

Nevertheless, the effects of postoperative in comparison to preoperative administration of morphine and phenylbutazone on horses’ recovery quality and postoperative behavior have not been studied yet.

The objective of this study was to determine if skin incision induces a modification of respiratory variables: inspiratory flow rate, tidal volume, and respiratory frequency in anesthetized horses undergoing bilateral fetlock arthroscopy. We also investigated if the preoperative (compared to postoperative) administration of morphine and phenylbutazone affect this response, recovery quality, postoperative behavior, and cardiorespiratory variables.

We tested the hypotheses that the breathing response to skin incision would be an increase in tidal volume and inspiratory flow rate without changes in respiratory frequency. We also hypothesized that the horses receiving morphine and phenylbutazone before surgery would have better recovery qualities and would show less postoperative behavioral changes in favor of pain than those receiving these analgesics after surgery.

## Materials and Methods

An abbreviation list is provided in Datasheet S1 in Supplementary Material.

This trial was a part of another study of the Equine Department and was performed in accordance with the EUROGUIDE on the accommodation and care of animals used for experimental and other scientific purposes published by the Royal Society of Medicine Press Limited (London, UK). The experimental procedure was approved by the Animal Ethics Committee of VetAgro-Sup (veterinary campus of Lyon), France (RECH-ETIC-P003-E01).

### Animals

Ten healthy (American Society of Anesthesiologists physical status score, ASA I) Standardbred horses (seven geldings and three mares), age (6.57 ± 1.27 years), weight (527 kg ± 49 kg) dedicated to research were included in the study.

Horses were randomly allocated to groups PRE (preoperative analgesia) and POST (postoperative analgesia) by simple randomization, using sequentially numbered, opaque, and sealed envelopes. Group PRE (*n* = 5) received morphine (Morphine Clorhydrate Aguettant, Aguettant Laboratory, France) 0.1 mg kg^−1^ and phenylbutazone (Phenylarthrite, Vétoquinol, France) 2.2 mg kg^−1^ IV immediately after induction of anesthesia (T0) and group POST *(n* = 5) received morphine and phenylbutazone at the end of the surgical procedure (last skin suture). The same volume of saline was administered in group POST at induction and in group PRE at recovery. The anesthetist was blinded to the treatment.

### Study Design

**Figure 1:**
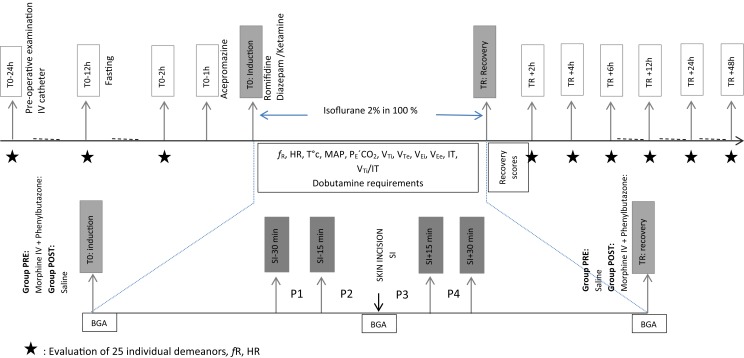
**Study design**.

The day before surgery, a 14-G catheter (Angiocath, Becton Dickinson, UT, USA) was inserted percutaneously into the right jugular vein. All the animals were fasted 12 h before anesthesia (Figure 1).

Acepromazine (Calmivet, Vetoquinol, France) 0.04 mg kg^−1^ was administered intramuscularly (IM) 1 h before induction of general anesthesia. In the induction box, all the horses were sedated with romifidine (Sedivet Boehringer Ingelheim, France) 0.04 mg kg^−1^ intravenously and anesthesia was induced with diazepam (Valium Roche, France) 0.05 mg kg^−1^ IV followed by ketamine (Imalgène1000, Merial, France) 2.2 mg kg^−1^ IV. A silicone endotracheal tube (outer diameter 26 mm) was introduced into the trachea and the cuff was inflated with a manometer pump to reach a pressure of 95 cmH_2_O to ensure a sealed airway. Horses were transferred to the operating room, placed in dorsal recumbency, and instrumented for monitoring. Ringer’s Lactate (Ringer Lactate Aguettant, Aguettant Laboratories, France) was administered at 10 mL kg^−1^ h^−1^.

Anesthesia was maintained with a 2% inspired fraction of isoflurane (F_I_′ISO) set on the vaporizer (F_I_′ISO: PRE = 2.0% [1.3, 2.4], POST = 1.9% [1.2, 2.2]; expired fraction of isoflurane, F_E_′ISO: PRE = 1.6% [1.3, 2], POST = 1.5% [1.2, 1.7]) (Isoflo, Abbot Laboratory, UK) in 100% O_2_. The horses were allowed to breathe spontaneously. The endpoints of the study were toxic signs of hypercapnia (mainly arrhythmias, hypoxemia) or a PaCO_2_ > 100 mmHg. In this case, horses would have been mechanically ventilated and withdrawn from the study.

Bilateral fetlock arthroscopy was performed by the same surgeon in all horses and anesthesia time was recorded.

### Cardiorespiratory Variables

Heart rate (HR, from the electrocardiogram), *f*_R_, and mean arterial blood pressure (MAP) from an arterial 20-G catheter (Insyte-W, Becton Dickinson, UT, USA) placed into the facial artery, body core temperature [T (°C)], peripheral arterial hemoglobin oxygen saturation (by pulse oximetry), end-tidal carbon dioxide tension (P_E_′CO_2_), inspired (_i_) and expired (_e_) *V*_T_ and minute volume $\left({\dot{V}}_{\text{E}}\right)$, and inspiratory time (IT) were recorded every 5 min with a previously calibrated monitor (Datex S/5, Datex-Ohmeda, GE Healthcare, Helsinki, Finland) and a flow sensor (Horse-Lite, Morpheus engineering, Wenum Wiesel, Netherlands). Monitored *V*_T_ and ${\dot{V}}_{\text{E}}$ values were multiplied by six following the manufacturer’s recommendations (17). The pressure transducer was zeroed against the atmospheric pressure and leveled at the height of the scapulohumeral joint. The inspiratory gas flow rate (*V*_Ti_/IT) was calculated.

### Dobutamine Requirements

Dobutamine (Dobutamine Panpharma 250 mg, Panpharma, France) IV was administered with a syringe driver (Syramed μSP6000, Arcomed, Switzerland) at a starting dose rate of 2 μg kg^−1^ min^−1^ when MAP decreased below 60 mmHg. Every 5 min, the dobutamine rate of infusion was increased by 2 μg kg^−1^ min^−1^ if the target MAP was not achieved, or stopped at any moment, once MAP reached 60 mmHg. The total amount of dobutamine per period was calculated for each horse and then averaged for each group.

### Rescue Doses of Thiopental

The depth of anesthesia was considered adequate when palpebral and corneal reflexes were present, the eye was central, with no presence of nystagmus. Light planes of anesthesia (in case of nystagmus or movement) were treated with thiopental (Nesdonal, Merial, France) 0.5 mg kg^−1^ IV. The number of boli was recorded.

### Blood–Gas Analysis

Three blood–gas analyses were performed (VetStat Idexx, France) to measure the pH and the arterial oxygen and carbon dioxide tensions (PaO_2_ and PaCO_2_, respectively). Analyses were performed once when the horse was instrumented, once when skin incision was performed, and at the end of the procedure (TR).

### Recovery Scores and Characteristics

Once the surgery was finished, the horses were transferred to the recovery box (TR) and were allowed to recover without assistance. Oxygen was provided by insufflation to all horses through a nasal tube (15 L min^−1^). Recoveries were video recorded and assessed by three experienced anesthetists who were unaware of the treatment.

**Figure 2:**
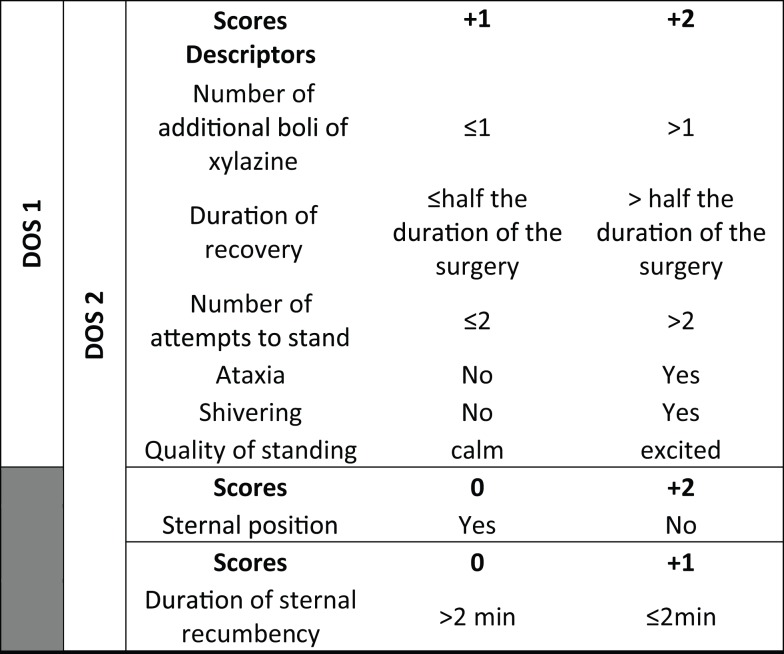
**Dichotomous objective scores**.

Recoveries were assessed with three scoring systems: a visual analog scale (RVAS: 0 cm: worst possible recovery and 10 cm: best possible recovery), a dichotomous subjective scale (DSS: good versus bad recovery), and a dichotomous objective score (DOS) (18) (Figure 2). Dichotomous objective descriptors (DODs) were used for objective scoring of recovery. DOSs (DOS1 and DOS2) were created compiling all the DODs (in DOS2) or rejecting the DODs “sternal position” and “duration of sternal position” (in DOS1). Descriptive objective scores for DOS 1 and DOS2 were calculated adding the scores mentioned in the table. For DOS2, the minimal possible score is 6 and the maximal possible score is 14. For DOS1, the minimal possible score is 6 and maximal possible score is 12. Contrary to the VAS, high DOS scores show bad recoveries and low DOS scores reveal good recoveries.

In addition to the recovery scores, the following recovery characteristics were analyzed: the time from placement in the recovery box to the time the horse was standing (RT), the time until the horse first moved (MOV), the time from the horses’ first movement until it was standing (MovUp), the number of tries (Tries), and the agitation index (AI), which is the ratio of tries and the time from the first movement to the horse standing. Xylazine boluses (0.1 mg kg^−1^) were prepared in advance in case the horse became dangerously agitated.

### Pre- and Postoperative Behavior

**Figure 3:**
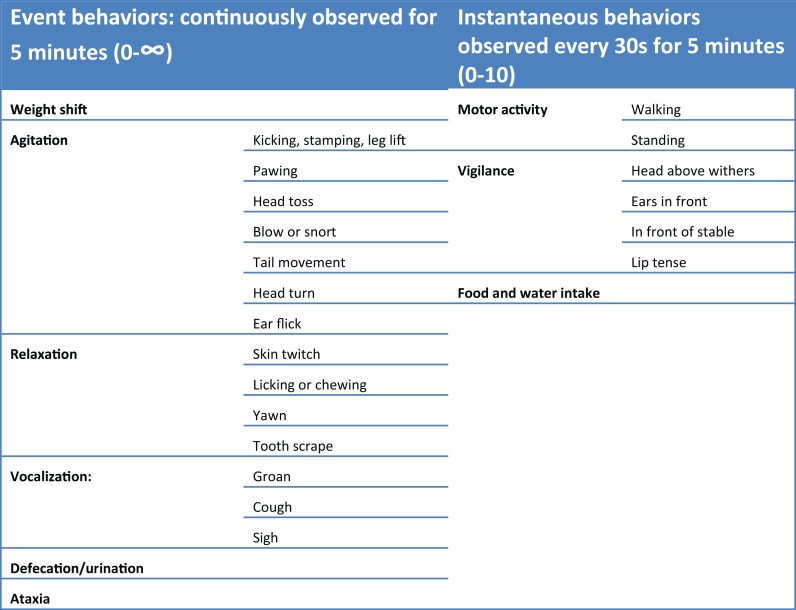
**Pre and post-operative behavior evaluation**.

Preanesthetic behaviors (baseline) were assessed at three time points, corresponding to 24, 12, and 2 h before anesthesia induction (T0–24 h, T0–12 h, and T0–2 h, respectively) by an observer blinded to the treatment from outside the box. After recovery from general anesthesia, all the horses returned to their boxes and 2 h were allowed for their acclimatization. Postoperative behaviors were assessed at 2, 4, 6, 12, 24, and 48 h after recovery (TR + 2 h, TR + 4 h, TR + 6 h, TR + 12 h, TR + 24 h, and TR + 48 h, respectively). Twenty-five individual demeanors were assessed separately. We have classified them into nine categories to facilitate the presentation of the results (Figure 3). Event behaviors were continuously observed for 5 min, the possible values obtained were between 0 and the infinity. Instantaneous behaviors were observed every 30 s for 5 min, which allowed a maximum score of 10 per descriptor.

Additionally to the behavior assessment, HR and *f*_R_ were measured at each time point.

### Statistical Analysis

Statistical analysis was performed with R Statistical software [R Core Team (19)]. Due to the small samples size, non-parametric tests were used.

#### Data Recorded During Surgery

Data were averaged within four periods: from 30 to 15 min before skin incision (P1), from 15 min to time of skin incision (P2), from skin incision to 15 min after incision (P3), and from 15 to 30 min after skin incision (P4).

A Wilcoxon rank-sum test was used to compare data between groups at the same period. A Wilcoxon signed-rank test was used to compare data at different periods within groups. Differences were considered significant if *p* was <0.05 unless a Bonferroni correction was applied (when more than two comparisons were performed) (*p* < 0.01). Data are presented as median [minimum, maximum].

#### Data Recorded During Recovery and the Postoperative Period

Treatment and time interactions were assessed using a Friedman test. A Wilcoxon signed-rank test or sum test was then used for comparison within or between groups.

Data are presented as median [minimum, maximum].

## Results

### Anesthesia Time and Rescue Doses of Thiopental

The anesthesia time was not significantly different between groups and was 115 [66, 120] min in PRE and 107 [52, 131] min in POST. Mean time for incision was not significantly different between groups and was 61 [54, 65] min in PRE and 57 [52, 71] min in POST.

Only two horses presented some nystagmus in group POST and required thiopental. The total number of boluses was two and four, respectively and they were administered after induction of general anesthesia, while the horses were being transported to the surgical table.

### Cardiorespiratory Variables

Respiratory rate, ${\dot{V}}_{\text{E}}$, IT, and *V*_Ti_/IT did not differ within or between groups (Table 1).

**Table 1:** **Cardiorespiratory variables, arterial blood–gas analysis, and dobutamine requirements in 10 isoflurane-anesthetized horses undergoing bilateral fetlock arthroscopy**.

Variable		PRE	POST
*f*_R_ (breaths min^−1^)	P1	3 [1, 11]	6 [1, 9]
	P2	3 [2, 8]	5 [1, 7]
	P3	4 [3, 7]	4 [3, 7]
	P4	4 [2, 5]	4 [2, 5]
*V*_Ti_ (mL kg^−1^)	P1	14 [10, 15]	9 [8, 14]
	P2	14 [11, 15]	11 [9, 13]^a^
	P3	16 [9, 20]	11 [9, 13]
	P4	16 [10, 21]	12 [9, 13]
*V*_Te_ (mL kg^−1^)	P1	14 [8, 24]	8 [8, 14]
	P2	13 [9, 15]	9 [8, 9]^a^
	P3	14 [8, 19]	10 [8, 11]
	P4	15 [10, 21]	12 [8, 13]
IT (s)	P1	3 [1, 4]	2 [1, 3]
	P2	3 [1, 5]	3 [2, 4]
	P3	3 [2, 6]	3 [2, 8]
	P4	3 [2, 6]	4 [2, 6]
${\dot{V}}_{\text{Ei}}$ (mL kg^−1^ min^−1^)	P1	51 [24, 10]	62 [14 76]
	P2	64 [31, 85]	61 [60, 66]
	P3	61 [39, 69]	55 [22, 68]
	P4	48 [47, 86]	33 [12, 48]
${\dot{V}}_{\text{Ee}}$ (mL kg^−1^ min^−1^)	P1	46 [24, 90]	54 [14, 73]
	P2	59 [30, 70]	49 [40, 58]
	P3	59 [37, 64]	53 [21, 55]
	P4	46 [44, 89]	32 [13, 47]
*V*_Ti_/IT (mL kg^−1^ s^−1^)	P1	3 [1, 3]	5 [1, 5]
	P2	4 [2, 6]	5 [5, 5]
	P3	4 [2, 8]	5 [2, 5]
	P4	6 [2, 8]	3 [1, 4]
P_E_′CO_2_ (mmHg)	P1	41 [31, 48]	40 [33, 50]
	P2	43 [38, 54]	45 [40, 46]
	P3	45 [37, 56]	44 [38, 50]
	P4	47 [39, 49]	40 [37, 53]
HR (beats min^−1^)	P1	41 [31, 48]	40 [33, 50]
	P2	43 [38, 54]	45 [40, 46]
	P3	45 [37, 46]	44 [38, 50]
	P4	47 [39, 49]	40 [37, 43]
MAP (mmHg)	P1	54 [50, 60]	61 [51, 62]
	P2	56 [53, 59]	61 [57, 77]
	P3	64 [53, 58]	69 [59, 88]
	P4	73 [62, 76]	63 [62, 85]
Dobutamine requirements by period (μg kg^−1^)	P1	18 [9, 36]	15 [6, 33]
	P2	18 [15, 27]	15 [0, 24]
	P3	9 [0, 21]	3 [0, 3]
	P4	0 [0, 6]	6 [0, 15]
T (°C)	P1	35.0 [34.8, 36.6]	35.4 [34.7, 36.2]
	P2	34.9 [34.4, 36.3]	35.1 [34.6, 36.2]
	P3	34.4 [33.9, 36.1]	34.8 [34.4, 35.8]
	P4	34.1 [33.5, 36.0]	34.7 [34.2, 35.4]
pH	G1	7.35 [7.27, 7.37]	7.33 [7.31, 7.35]
	G2	7.28 [7.22, 7.36]	7.28 [7.20, 7.33]
	G3	7.29 [7.32, 7.23]	7.29 [7.19, 7.30]
PaCO_2_ (mmHg)	G1	56 [48, 76]	56 [55, 66]
	G2	73 [61, 75]	66 [60, 83]
	G3	75 [69, 85]	71 [60, 91]
PaO_2_ (mmHg)	G1	322 [74, 399]	356 [284, 442]
	G2	186 [171, 287]	254 [135, 391]
	G3	108 [72, 148]	125 [74, 285]

*Horses received morphine and phenylbutazone before skin incision (PRE, *n* = 5) or at the end of the procedure (POST, *n* = 5). Data are reported as median [min, max]. *f*_R_, respiratory frequency; *V*_Ti_, inspired tidal volume; *V*_Te_, expired tidal volume; ${\dot{V}}_{\text{Ei}}$, inspired minute volume; ${\dot{V}}_{\text{Ee}}$, expired minute volume; *V*_Ti_/IT, inspiratory flow rate; P_E_CO_2_, expired pressure of carbon dioxide; HR, heart rate; MAP, mean arterial blood pressure; PaCO_2_, arterial partial pressure of carbon dioxide; PaO_2_, arterial partial pressure of oxygen; P1, from 30 to 15 min before skin incision; P2, from 15 min to time of skin incision; P3, from skin incision to 15 min after incision; P4 from 15 to 30 min after skin incision. Arterial gas analysis periods: G1, once when the horse was instrumented, G2, once when skin incision was performed, and G3, at the end of the procedure. Data are reported as median [min, max]*.

*^a^Indicates statistically significant differences between groups (*p* < 0.05)*.

**Figure 4:**
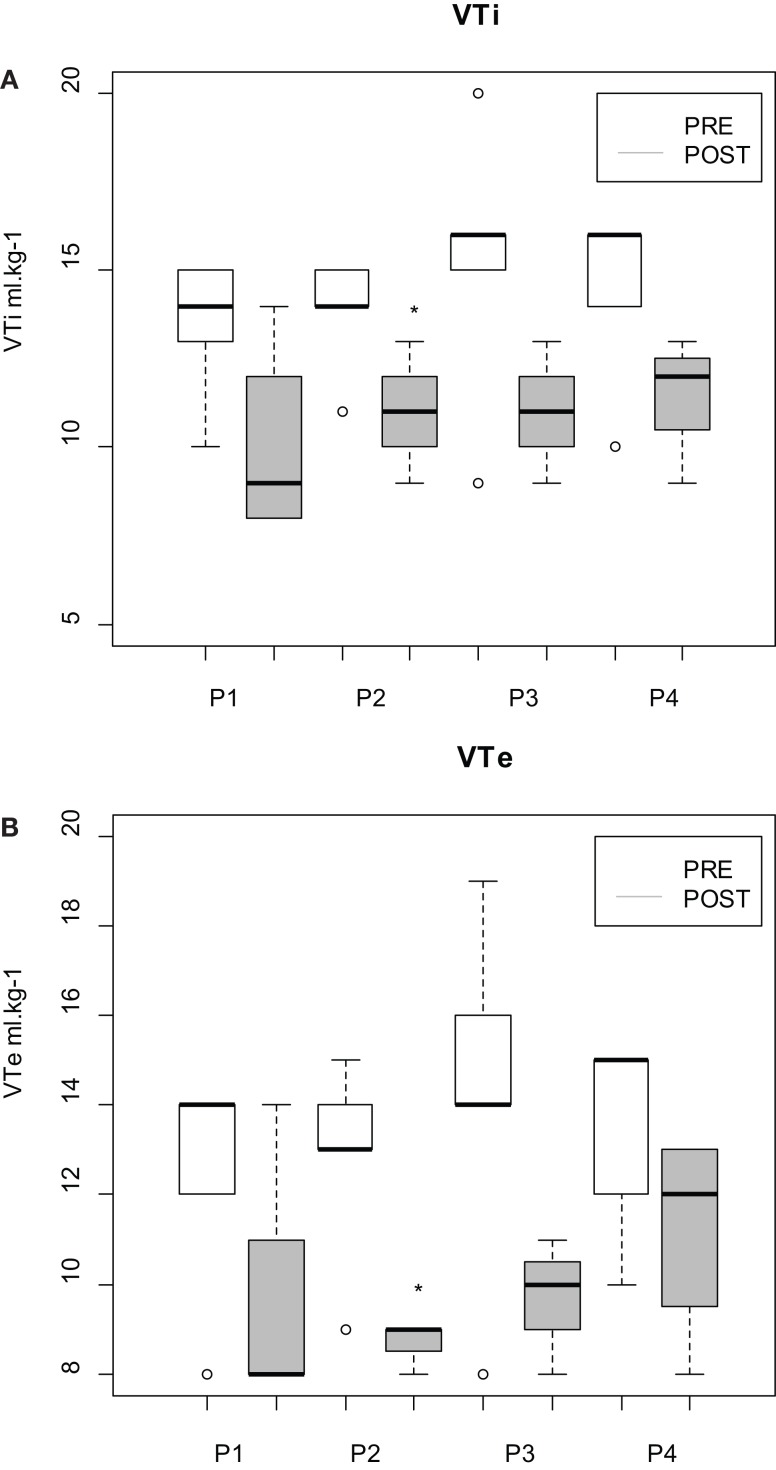
**(A)** Inspired (_i_) and **(B)** expired (_e_) tidal volume (*V*_T_) measured in isoflurane-anesthetized horses undergoing fetlock arthroscopy. Horses received morphine and phenylbutazone before skin incision (PRE) or at the end of the procedure (POST). Four periods of time were defined: P1, from 30 to 15 min before skin incision; P2, from −15 min to skin incision; P3, from skin incision to 15 min after incision; and P4, from 15 to 30 min after skin incision. The boxplots represent the first and third quartile (Q1 and Q3, respectively) and the median. The lower dispersion bar represents, in the distribution, the value which is immediately superior to the adjacent value [Q1 − 1.5(Q3 − Q1)]. The higher dispersion bar represents, in the distribution, the value which is immediately inferior to the adjacent value [Q1 + 1.5(Q3 − Q1)]. *Indicates statistically significant differences between groups (*p* < 0.05).

During all the periods, *V*_Te_ and *V*_Ti_ were higher in PRE than in POST. Nevertheless, the difference was significant only during P2 (*p* = 0.01) (Figure 4).

Heart rate and MAP did not differ within or between groups.

### Dobutamine Requirements

There was a tendency, in horses allocated to group POST, to receive less dobutamine from P2 to P3. However, this difference was not significant when the Bonferroni correction was applied (*p* = 0.03) (Table 1).

### Blood–Gas Analysis

Arterial pH, PaO_2_, and PaCO_2_ did not differ within or between groups (Table 1).

### Recovery Scores and Characteristics

No significant differences between groups were found in any of the recovery scores or characteristics. No horse received xylazine (Table 2).

**Table 2:** **Recovery scores and characteristics in 10 isoflurane-anesthetized horses undergoing bilateral fetlock arthroscopy**.

	PRE	POST
RVAS	3.8 [3.7–9.2]	4.4 [3.1–8.5]
DSS	2 [1–2]	2 [1–2]
DOS 1	9 [8–10]	9 [8–10]
DOS 2	10 [8–11]	9 [8–10]
RT (min)	17 [4–52]	16 [5–22]
Mov (min)	4 [1–24]	6 [1–13]
MovUp (min)	12 [3–2]	9 [3–10]
Tries	3 [1–6]	7 [2–10]
AI	0.3 [0.04–2]	0.9 [0.22–2.3]

*Horses received morphine and phenylbutazone before skin incision (PRE, *n* = 5) or at the end of the procedure (POST, *n* = 5). A visual analog scale (RVAS), a dichotomous subjective scale (DSS), a dichotomous objective score (DOS), the time from placement in the recovery box to the time the horse was standing (RT), the time until the horse first moved (MOV), the time from the horse’s first movement until it was standing (MovUp), the number of tries (Tries), and the agitation index (AI) were calculated. Data are reported as median [min, max]*.

### Pre- and Postoperative Behavior

Concerning the behavior assessment, horses in PRE spent significantly more time walking at T0 + 2 h than in POST (*p* = 0.02).

No other significant difference was found in any of the nine categories. No significant differences were identified in HR or *f*_R_ between the groups.

These results are detailed in Datasheet S2 in Supplementary Material.

## Discussion

In this study, skin incision did not modify respiratory variables in horses undergoing fetlock arthroscopy under general anesthesia in dorsal recumbency. Horses that received morphine and phenylbutazone preoperatively had a higher *V*_T_ but the same *f*_R_ than horses that received the same treatment postoperatively. The quality of recovery was the same whether morphine and phenylbutazone were administered before or after the surgery. Horses also showed few postoperative behavioral differences between groups.

A needle prick is considered to be a high-intensity, noxious stimulus, leading to an adaptive and protective reaction, due to a brief and localized signal of potential tissue damage (20). Therefore, we expected all the more that skin incision evokes somatic nociception. Surgical incision has been used as a nociceptive stimulus in former (5, 7, 8) as well as in more recent (21–23) studies in man. In animals, we found only one clinical study using skin incision preceding castration as a model of nociception (24).

However, in our study, this stimulus did not seem to produce nociception. MAP seems to be the most sensitive and reliable indicator of nociception in isoflurane-anesthetized horses (24). In the present study, skin incision did not affect this variable (even in group POST) and might, therefore, be considered as an insufficient nociceptive stimulus.

Heart rate and MAP were mainly higher than 40 bpm and lower than 70 mmHg, respectively, which could have reflected deep plane of anesthesia that, in turn, may have interfered with the recognition of nociception. However, this is unlikely because a palpebral reflex was maintained during the whole procedure confirming appropriate or even light stage of anesthesia.

The clinical properties of the drugs used for premedication and induction of general anesthesia may also have altered the nociceptive effects of skin incision. Nevertheless, premedication with alpha-2 agonist is mandatory before induction of anesthesia in horses. All alpha-2 agonists present analgesic properties, and therefore, it would have been difficult to draw up a protocol without analgesic drugs. The analgesic effects of romifidine after IV injection may last 120 min (25), and in our study, the mean time for incision from induction was 60 min (26, 27). Ketamine half-life is 40–60 min. It is, therefore, possible that romifidine and ketamine had an effect on the response to skin incision. However, in the study by Haga and Dolvik (24), alpha-2 agonist (detomidine) and ketamine did not conceal the nociceptive effect of surgery when administered to horses for premedication and induction of anesthesia respectively. Furthermore, the response to skin incision was not suppressed in men that had not received any premedication but a constant rate infusion (CRI) of opioid throughout the procedure (8).

A study in deeply anesthetized rabbits showed that low- and high-frequency tooth pulp stimulation (tooth afferent fibers almost exclusively provides nociceptive information) evoked respiratory response (increased ${\dot{V}}_{\text{E}}$ and *f*_R_) only when it was associated with muscular contractions of the digastric muscle (28). This suggests that noxious stimulus could only affect respiratory variables when ergoreceptors are stimulated by muscular contraction. In our study, incision was performed at the fetlock level and did not involve muscular fibers or muscular contraction. Abdominal incision during colic surgery, for example, could be a more accurate nociceptive stimulus.

An increase in *V*_T_ and *V*_Ti_/IT in group POST was expected following skin incision. Previous studies in human patients showed that noxious stimulus affects the drive (represented by *V*_Ti_/IT) and the timing of respiration (inspiratory and expiratory times) (7, 8). The mean inspiratory flow rate indicates the rate of increase in central inspiratory neural activity and is, therefore, a good indicator of central neural output. The duration of the phases of the respiratory cycle (IT) reflects the central control and the pulmonary and somatic afferent influences. In anesthetized human patients, some studies demonstrated that noxious stimulus induces an increase in respiratory drive and a decrease (6) or no change (7) in respiratory timing. These aspects of the control of breathing may, on one hand, be affected by opioids that may reduce the drive and increase expiration time (29) and, on the other hand, by the noxious stimulus that may reduce these effects (8). However, it is not possible to comment on how skin incision affected the control of breathing in the present study as skin incision did not seem to produce nociception.

Nevertheless, it is interesting to note that *V*_Te_ and *V*_Ti_ were overall higher in PRE than in POST. Among the analgesic drugs used in PRE, phenylbutazone does not seem to affect cardiorespiratory variables (12), but morphine may stimulate pulmonary ventilation via central nervous excitation (30). Nevertheless, it is unlikely to occur under anesthesia. In man, opioids are rather known to induce respiratory depression (31). Morphine decreased minute volume by 10.3% in patients suffering from acute postoperative pain (32). However, we found little information in the literature about the effects of morphine on respiratory volumes in anesthetized animals. Nolan et al. (11) found that anesthetized horses, receiving an IV bolus of 0.05 mg kg^−1^ morphine, had significantly higher ${\dot{V}}_{\text{E}}$ compared with controls. Nevertheless, *V*_T_ and PaCO_2_ were not significantly different between groups and higher ${\dot{V}}_{\text{E}}$ resulted from lower *f*_R_ in the saline group. In addition, in the morphine group, ${\dot{V}}_{\text{E}}$, *V*_T_, and PaCO_2_ were not significantly different before and after the injection of the drug. Although breathing volumes were not measured, other authors studied the effect of a bolus of morphine on respiratory variables. Arterial carbon dioxide tension was not modified by a bolus of morphine (0.25 mg kg^−1^) administered to horses anesthetized with isoflurane in oxygen (33). In the same study, a higher dose of morphine (2 mg kg^−1^) induced respiratory depression objectified by increased PaCO_2_. Mircica et al. (34) also reports minimal respiratory effects of low clinical doses of morphine (0.1–0.17 mg kg^−1^) in the halothane-anesthetized horse.

In our study, it remains unclear why horses treated with morphine and phenylbutazone at induction had higher *V*_T_ than horses that received the drugs postoperatively unless a greater inspired concentration of isoflurane was administered in order to maintain surgical depth of anesthesia in group POST. Isoflurane has indeed been shown to produce respiratory depression (35). This is unlikely because F_I_′ISO and F_E_′ISO were not significantly different in both groups. In addition, respiratory frequency did not differ between groups. This is in agreement with other studies (15, 34, 36) that demonstrated that morphine has no effect on *f*_R_ and arterial blood–gas analysis.

With regard to recovery quality and characteristics, although better recoveries were expected in PRE than in POST, significant differences were not observed between groups.

Preemptive analgesia refers to evidence that analgesic preoperative treatment is more effective than the same treatment administered after incision or surgery (37). Clark (13) reviewed both the human and veterinary literature on this subject. The author concluded that the evidence pertaining to preemptive analgesia is equivocal and not convincing in human literature. In veterinary medicine, only one study in cats (38) met the criteria (same pre- and postintervention) of this concept. The effects of preemptive analgesia on the quality of recovery from anesthesia in horses are not known. Nevertheless, some authors compared the postoperative effect of some analgesics given preoperatively with a placebo. Although there is no strong evidence in the literature that phenylbutazone can adversely affect recoveries, one study in horses undergoing arthroscopy showed that horses premedicated with phenylbutazone had slightly worse subjective recovery scores than controls with saline (4). The effects of morphine on the quality of recovery in equine anesthesia are also controversial, although there is stronger opinion to support its beneficial effects when used at low doses. Steffey et al. (33) considered that recovery from anesthesia after administration of high dose of morphine (2 mg kg^−1^) was dangerous, but horses recovered reasonably well after administration of a lower “clinical” dose (0.25 mg kg^−1^). Horses undergoing elective surgical procedure and receiving a bolus of morphine before induction of anesthesia and a CRI of morphine during anesthesia showed fewer attempts to attain sternal recumbency and standing [Clark et al. (16)] than horses that received the same volume of saline. Love et al. (15) also found better recovery subjective scores in horses treated with a bolus of morphine (0.1–0.2 mg kg^−1^) after induction than in controls. This study failed to show the beneficial effects of the preoperative analgesic treatment on the quality of recovery. The analgesic effects of the anesthetic molecules can have concealed the difference between both groups.

Postoperative behaviors and cardiorespiratory variables did not differ between groups. Some studies demonstrated more box walking or signs of discomfort and agitation in horses that received a morphine CRI during general anesthesia compared to dexmedetomidine CRI (39). As mentioned above, in the present study, skin incision followed by arthroscopy may be considered as an insufficient nociceptive stimulus. Arthroscopy was used as a model of postoperative pain behavior in several clinical studies (3, 4). In our study, horses had healthy joints and were pain free at presentation. Consequently, they may have experienced a lower degree of pain than horses presented with damaged joints.

The performance of this study is not free of other limitations. All horses received the antinociceptive treatment (whether pre- or postoperatively), which may have masked the effect of surgical stimulation on postoperative behaviors. Although unethical, a third group with no analgesia at all would have been interesting to show any effect of central sensitization caused by inflammatory injury (40).

The lack of effect of the surgical stimulus on cardiorespiratory variables may also result from the insufficient sample size. We were not able to control the sample size because, in order to respect the three Rs of animal experimentation, we chose to benefit from another clinical study realized on horses in our faculty. A *post hoc* sample size was calculated on the basis of *V*_T_, showing that 19 horses would have been necessary in each group to get a 90% chance to detect, with a risk of 5%, an average difference of *V*_T_ of 1 L between the two groups, considering a common SD of 1.3 L.

## Conclusion

With the protocol used in this study and the number of horses included, it is difficult to conclude if tidal volume and respiratory frequency can be used as markers of nociception in horses because skin incision did not induce nociception in this study. For further experimental studies, higher sample size, anesthetic drugs without analgesic properties or more efficient nociceptive stimulus, and pain markers should be used. Nevertheless, this pilot study delivers important information for prospective future trials.

## Author Contributions

Participated in research design: CCR, ICCB, and KP. Conducted experiments: CCR, ICCB, IG, and KP. Performed data analysis: CCR, ICCB, IG, and KP. Wrote or contributed to the writing of the manuscript: CCR, ICCB, and KP. Revised the manuscript: CCR, ICCB, IG, KP, and SG.

## Conflict of Interest Statement

The authors declare that the research was conducted in the absence of any commercial or financial relationships that could be construed as a potential conflict of interest.
