# Supplementary material for: Effect of Pre- and Postoperative Phenylbutazone and Morphine Administration on the Breathing Response to Skin Incision, Recovery Quality, Behavior, and Cardiorespiratory Variables in Horses Undergoing Fetlock Arthroscopy: A Pilot Study
Source: Front Vet Sci. 2015 Nov 23;2:58. doi: 10.3389/fvets.2015.00058 (PMC4672197; doi:10.3389/fvets.2015.00058)
Supplement: Supplementary file 1 [file Data_Sheet_1.DOCX]

AI: agitation index which is the ratio of tries and the time from the first movement to the horse standing

DOS: dichotomous objective score

DSS: dichotomous subjective scale

F_E_’ISO: expired fraction of isoflurane measured by IR spectrophotometry at the Y piece level

F_I_’ ISO: inspired fraction of isoflurane measured by IR spectrophotometry at the Y piece level

*f*_R_: Respiratory rate

HR: Heart rate

IM: Intramuscularly

IT: Inspiration time

IV: Intravenously

MAP: Mean arterial blood pressure

MOV: the time from placement in the recovery box (TR) until the horse first moved

MovUp: the time from the horses’ first movement (MOV) until it was standing

PaCO2: arterial carbon dioxide tension

PaO2: arterial oxygen tensión

P_E_’CO_2_: end-tidal carbon dioxide tension

POST: group of horses that received morphine and phenylbutazone immediately post surgery

PRE: group of horses that received morphine and phenylbutazone immediately post induction time.

RT: recovery time: the time from placement in the recovery box (TR) to the time the horse was standing

RVAS: Recovery visual analogue scale

T°C: body core temperature

T0: Induction time

TR: Recovery point (time when the horses were placed in the recovery box)

Tries: the number of tries

.

.

V̇_E_: Minute volume

V̇_Ei_: inspired minute volume

V̇_Ee_: expired minute volume

V_Ti_/ IT: Inspiratory flow rate

V_T_: Tidal volume

V_Te_: Expired tidal volume

V_Ti_: Inspired tidal volume
