# Supplementary material for: Effect of Pre- and Postoperative Phenylbutazone and Morphine Administration on the Breathing Response to Skin Incision, Recovery Quality, Behavior, and Cardiorespiratory Variables in Horses Undergoing Fetlock Arthroscopy: A Pilot Study
Source: Front Vet Sci. 2015 Nov 23;2:58. doi: 10.3389/fvets.2015.00058 (PMC4672197; doi:10.3389/fvets.2015.00058)
Supplement: Supplementary file 2 [file Data_Sheet_2.DOCX]

**Appendix 2.**

Post-operative behavior frequency in ten isoflurane anesthetized horses undergoing bilateral fetlock arthroscopy. Horses received morphine and phenylbutazone before skin incision (PRE, *n* = 5) or at the end of the procedure (POST, *n* = 5). Behaviors were assessed at three time points, T1,T2 and T3 corresponding to 24, 12, and 2h before anesthesia induction and at 6 time points, T4-T9 corresponding to 2, 4, 6, 12, 24 and 48h after recovery. Data are reported as median [min, max]. * Indicates statistically significant differences between groups (p < 0.05).

| **DEMEANOURS/HR/*f*R** | Group | T1 | T2 | T3 | T4 | T5 | T6 | T7 | T8 | T9 |
| --- | --- | --- | --- | --- | --- | --- | --- | --- | --- | --- |
| **HR** (**beats minute^-1^**) | PRE | 44 [36,52] | 33 [32,52] | 40 [36,44] | 52 [38,60] | 44 [30,48] | 44 [36,54] | 42 [40,48] | 48 [36,53] | 30 [36,44] |
|  | POST | 48 [44,56] | 40 [34,50] | 40 [36,60] | 44 [40,56] | 40 [36,60] | 40 [44,48] | 42 [28,56] | 44 [36,52] | 36 [30,44] |
| ***f*R** (**breaths minute^-1^**) | PRE | 12 [12,18] | 12 [10,16] | 12 [10,16] | 12 [10,24] | 08 [14,16] | 08 [12,16] | 08 [12,16] | 8 [10,16] | 10 [08,16] |
|  | POST | 12 [12,18] | 12 [08,16] | 14 [12,16] | 12 [08,12] | 16 [08,16] | 12 [08,14] | 12 [06,16] | 12 [08,16] | 10 [08,16] |
| **Weight shift** (number observed continuously observed for 5 minutes) | PRE | 00 [00,02] | 00 [02,06] | 00 [00,02] | 02 [00,04] | 02 [00,04] | 02 [00,05] | 02 [00,03] | 00 [00,02] | 01 [00,08] |
|  | POST | 01 [00,02] | 02 [00,06] | 01 [00,06] | 01 [00,03] | 00 [00,04] | 00 [00,07] | 01 [00,02] | 00 [00,13] | 01 [00,08] |
| **Urination** (number observed continuously observed for 5 minutes) | PRE | 00 [00,00] | 00 [00,00] | 00 [00,01] | 00 [00,01] | 00 [00,00] | 00 [00,00] | 00 [00,00] | 00 [00,00] | 00 [00,00] |
|  | POST | 00 [00,00] | 00 [00,00] | 00 [00,01] | 00 [00,00] | 00 [00,00] | 00 [00,00] | 00 [00,00] | 00 [00,00] | 00 [00,00] |
| **Defecation** (number observed continuously observed for 5 minutes) | PRE | 00 [00,01] | 00 [00,00] | 00 [00,00] | 00 [00,00] | 00 [00,01] | 00 [00,00] | 00 [00,00] | 00 [00,00] | 00 [00,00] |
|  | POST | 00 [00,00] | 00 [00,00] | 00 [00,00] | 00 [00,00] | 00 [00,00] | 00 [00,00] | 00 [00,00] | 00 [00,00] | 00 [00,00] |
| **Ataxia** (number observed continuously observed for 5 minutes) | PRE | 00 [00,00] | 00 [00,00] | 00 [00,00] | 00 [00,04] | 00 [00,02] | 00 [00,00] | 00 [00,00] | 00 [00,00] | 00 [00,00] |
|  | POST | 00 [00,02] | 00 [00,00] | 00 [00,00] | 00 [00,00] | 00 [00,00] | 00 [00,00] | 00 [00,00] | 00 [00,00] | 00 [00,00] |
| **Food and water intake** (number observed every 30 seconds for 5 minutes) | PRE | 02 [00,08] | 01 [00,10] | 06 [02,07] | 02 [00,08] | 01 [00,10] | 05 [00,09] | 08 [03,10] | 04 [00,10] | 08 [00,08] |
|  | POST | 4.5 [00,08] | 03 [00,10] | 04 [00,08] | 02 [00,10] | 07 [00,10] | 08 [00,10] | 04 [00,10] | 01 [00,10] | 08 [00,08] |
| **AGITATION** (number observed continuously observed for 5 minutes) |  | | | | | | | | | |
| Kicking, stamping, leg lift | PRE | 01 [00,02] | 01 [00,04] | 00 [00,01] | 02 [00,02] | 02 [00,14] | 02 [00,51] | 00 [00,05] | 00 [00,03] | 01 [00,03] |
|  | POST | 01 [00,02] | 02 [00,04] | 00 [00,01] | 02 [00,04] | 02 [00,14] | 01 [00,03] | 01 [00,05] | 00 [00,01] | 01 [00,03] |
| Pawing | PRE | 00 [00,00] | 01 [00,06] | 00 [00,01] | 00 [00,03] | 00 [00,07] | 02 [00,06] | 07 [00,12] | 00 [00,01] | 00 [00,01] |
|  | POST | 00 [00,02] | 00 [00,02] | 00 [00,03] | 00 [00,06] | 00 [00,00] | 01 [00,07] | 00 [00,11] | 00 [00,03] | 00 [00,01] |
| Head toss | PRE | 00 [00,06] | 01 [00,01] | 02 [00,03] | 00 [00,05] | 03 [00,10] | 00 [00,01] | 00 [00,07] | 00 [00,00] | 00 [00,03] |
|  | POST | 0.5 [00,01] | 00 [00,01] | 01 [00,12] | 00 [00,01] | 00 [00,23] | 02 [00,02] | 00 [00,07] | 01 [00,04] | 00 [00,03] |
| Blow or Snort | PRE | 05 [01,20] | 05 [02,10] | 03 [01,15] | 01 [00,05] | 02 [00,07] | 01 [00,05] | 01 [00,02] | 01 [00,04] | 01 [00,15] |
|  | POST | 4.5 [02,08] | 01 [00,06] | 04 [00,15] | 01 [00,01] | 02 [00,03] | 01 [00,02] | 02 [00,02] | 00 [00,04] | 01 [00,15] |
| Tail movement | PRE | 01 [00,04] | 00 [00,14] | 00 [00,00] | 00 [00,01] | 00 [00,07] | 00 [00,08] | 00 [00,00] | 00 [00,00] | 00 [00,04] |
|  | POST | 00 [00,00] | 00 [00,00] | 00 [00,00] | 00 [00,05] | 00 [00,00] | 00 [00,02] | 00 [00,01] | 00 [00,00] | 00 [00,04] |
| Head Turn | PRE | 00 [00,00] | 00 [00,00] | 00 [00,00] | 00 [00,00] | 00 [00,00] | 00 [00,03] | 00 [00,00] | 00 [00,00] | 00 [00,00] |
|  | POST | 00 [00,00] | 00 [00,00] | 00 [00,00] | 00 [00,00] | 00 [00,00] | 00 [00,00] | 00 [00,00] | 00 [00,00] | 00 [00,00] |
| Ear Flick | PRE | 07 [05,26] | 05 [00,13] | 06 [03,27] | 08 [00,12] | 08 [00,21] | 08 [00,07] | 02 [00,07] | 00 [00,00] | 02 [01,09] |
|  | POST | 03 [01,05] | 10 [05,21] | 05 [01,20] | 01 [00,04] | 00 [00,02] | 01 [00,05] | 05 [00,18] | 10 [01,29] | 02 [01,09] |
| **RELAXATION** (number observed continuously observed for 5 minutes) |  | | | | | | | | | |
| Skin Twitch | PRE | 00 [00,01] | 00 [00,01] | 00 [00,00] | 01 [00,02] | 00 [00,01] | 00 [00,04] | 00 [00,06] | 00 [00,00] | 00 [00,02] |
|  | POST | 02 [00,04] | 00 [00,01] | 00 [00,00] | 00 [00,00] | 00 [00,02] | 00 [00,01] | 00 [00,06] | 00 [00,01] | 00 [00,02] |
| Licking and Chewing | PRE | 01 [00,03] | 01 [00,09] | 08 [05,11] | 03 [03,08] | 04 [01,11] | 05 [00,10] | 06 [03,10] | 00 [00,06] | 01 [00,02] |
|  | POST | 00 [00,03] | 02 [00,05] | 06 [01,08] | 11 [03,15] | 09 [01,14] | 06 [00,09] | 07 [01,12] | 01 [00,18] | 01 [00,02] |
| Yawn | PRE | 00 [00,00] | 00 [00,00] | 00 [00,00] | 00 [00,00] | 00 [00,00] | 00 [00,00] | 00 [00,00] | 00 [00,00] | 00 [00,00] |
|  | POST | 00 [00,00] | 00 [00,00] | 00 [00,00] | 00 [00,00] | 00 [00,00] | 00 [00,00] | 00 [00,00] | 00 [00,00] | 00 [00,00] |
| Tooth scrape | PRE | 00 [00,01] | 00 [00,01] | 00 [00,00] | 00 [00,00] | 00 [00,00] | 00 [00,00] | 00 [00,00] | 00 [00,00] | 00 [00,00] |
|  | POST | 00 [00,00] | 00 [00,03] | 00 [00,02] | 00 [00,00] | 00 [00,00] | 00 [00,00] | 00 [00,00] | 00 [00,08] | 00 [00,00] |
| **VOCALISATION** (number observed continuously observed for 5 minutes) | PRE | 00 [00,04] | 00 [00,01] | 00 [00,00] | 00 [00,00] | 00 [00,01] | 00 [00,01] | 00 [00,00] | 00 [00,03] | 00 [00,00] |
|  | POST | 00 [00,00] | 00 [00,01] | 00 [00,03] | 00 [00,00] | 00 [00,05] | 00 [00,00] | 00 [00,00] | 00 [00,01] | 00 [00,00] |
| Groan | PRE | 00 [00,00] | 00 [00,00] | 00 [00,00] | 00 [00,00] | 00 [00,00] | 00 [00,00] | 00 [00,00] | 00 [00,01] | 00 [00,01] |
|  | POST | 00 [00,00] | 00 [00,00] | 00 [00,00] | 00 [00,00] | 00 [00,00] | 00 [00,00] | 00 [00,02] | 00 [00,00] | 00 [00,01] |
| Cough | PRE | 00 [00,00] | 00 [00,00] | 00 [00,00] | 00 [00,00] | 00 [00,00] | 00 [00,01] | 00 [00,00] | 00 [00,00] | 00 [00,01] |
|  | POST | 00 [00,00] | 00 [00,00] | 00 [00,00] | 00 [00,00] | 00 [00,00] | 00 [00,00] | 00 [00,00] | 00 [00,00] | 00 [00,01] |
| Sigh | PRE | 00 [00,00] | 00 [00,00] | 00 [00,00] | 00 [00,00] | 00 [00,00] | 00 [00,00] | 00 [00,00] | 00 [00,00] | 00 [00,00] |
|  | POST | 00 [00,00] | 00 [00,00] | 00 [00,00] | 00 [00,00] | 00 [00,00] | 00 [00,00] | 00 [00,01] | 00 [00,00] | 00 [00,00] |
| **MOTOR ACTIVITY** (number observed every 30 seconds for 5 minutes) |  | | | | | | | | | |
| Standing | PRE | 10 [07,10] | 08 [09,10] | 09 [03,10] | 08 [07,10] | 09 [07,10] | 09 [07,10] | 09 [10,10] | 10 [07,10] | 08 [00,10] |
|  | POST | 9.5 [07,10] | 09 [08,10] | 09 [08,10] | 10 [10,10] | 10 [09,10] | 09 [09,10] | 10 [00,10] | 10 [10,10] | 08 [00,10] |
| Walking | PRE | 00 [00,03] | 01 [00,02] | 01 [00,07] | 02 [00,03]* | 01 [00,03] | 01 [00,03] | 00 [00,01] | 00 [00,03] | 02 [00,10] |
|  | POST | 0.5 [00,03] | 01 [00,02] | 01 [00,02] | 00 [00,00] | 00 [00,01] | 01 [00,01] | 00 [00,10] | 00 [00,00] | 02 [00,10] |
| **VIGILANCE** (number observed every 30 seconds for 5 minutes) |  | | | | | | | | | |
| Head above withers | PRE | 07 [05,10] | 06 [01,10] | 09 [03,10] | 05 [00,08] | 01 [00,10] | 01 [00,04] | 02 [00,08] | 06 [01,09] | 02 [00,00] |
|  | POST | 0.5 [00,03] | 06 [00,10] | 08 [02,09] | 00 [00,10] | 01 [00,05] | 01 [00,04] | 01 [00,10] | 08 [01,10] | 02 [00,08] |
| Ears in front | PRE | 09 [05,10] | 08 [01,10] | 10 [05,10] | 07 [02,09] | 04 [00,10] | 02 [00,10] | 02 [02,10] | 07 [04,10] | 04 [02,09] |
|  | POST | 07 [05,10] | 06 [00,10] | 06 [06,09] | 02 [00,10] | 02 [00,04] | 01 [00,05] | 00 [00,10] | 07 [01,09] | 04 [02,09] |
| Lip tense | PRE | 08 [02,10] | 09 [00,10] | 04 [03,08] | 07 [02,10] | 07 [00,09] | 01 [01,10] | 02 [00,07] | 06 [00,10] | 02 [02,10] |
|  | POST | 5.5 [02,10] | 07 [00,10] | 06 [02,07] | 07 [00,10] | 02 [00,10] | 02 [00,10] | 06 [00,10] | 09 [00,10] | 02 [02,10] |
| In front of stable | PRE | 09 [00,10] | 10 [08,10] | 10 [02,10] | 07 [01,08] | 10 [00,10] | 00 [00,08] | 04 [00,09] | 09 [00,10] | 08 [00,10] |
|  | POST | 08 [00,10] | 09 [08,10] | 09 [02,10] | 08 [00,10] | 00 [00,10] | 08 [00,10] | 10 [00,10] | 10 [00,10] | 00 [08,10] |
